# 1270. Molecular Characterization of Carbapenemase Producing Enterobacterales, *Acinetobacter* spp. and *Pseudomonas* spp. in Nosocomial and Community-acquired Clinical Isolates in Bogota, Colombia

**DOI:** 10.1093/ofid/ofab466.1462

**Published:** 2021-12-04

**Authors:** Luis F Reyes, Ingrid G Bustos-Moya, Diego Josa, Enrique Gamboa-Silva, Elsa Daniela Ibañez-Prada, Hector Africano, Juan Urrego-Reyes, Claudia Beltrán, Sebastian Leon, Alejandra Ruiz-Cuartas, Oscar Baron, Rafael Leal, Jane Hawkey, Kelly Wyres, Andrew Stewardson

**Affiliations:** 1 Universidad de La Sabana, Bogota, Distrito Capital de Bogota, Colombia; 2 Universidad de la Sabana, Cajica, Cundinamarca, Colombia; 3 Universidad de la Sabana, Chía, Colombia, Bogota, Cundinamarca, Colombia; 4 MSD Colombia, Bogota, Cundinamarca, Colombia; 5 MSD Colombia, Bogotá, Colombia, Bogotá, Distrito Capital de Bogota, Colombia; 6 Fundación Clínica Shaio, Bogota, Cundinamarca, Colombia; 7 Monash University, Melborne, Western Australia, Australia

## Abstract

**Background:**

Antimicrobial resistance (AMR) in low-income and middle-income countries (LMICs) is a public health problem. AMR is a concerning problem in Gram-negative bacteria such are Enterobacterales, which are frequently carbapenem-resistant pathogens (CRP), and few therapeutic options are available. However, scarce data is known regarding the clinical, molecular characteristics, and clinical outcomes of patients infected with carbapenem-resistant pathogens in LMICs. Thus, this study will attempt to bring novel data in these regards.

**Methods:**

This is a retrospective cohort study conducted in two reference hospitals in Colombia, South America. All consecutive patients infected with CRPs between 2017 and 2021 were included. Clinical data were gathered by retrospective chart review. Bacterial pathogens and antibiotic susceptibility were prospectively identified and stored by each hospital. Molecular characterization was performed by PCR in isolated bacteria.

**Results:**

A total of 220 patients were included. The mean (SD) age was 60.6 (18.4) years, and 32% (71/220) were female. The most frequently identified CRPs were *Pseudomonas aeruginosa* (85/220, 39%) and *Klebsiella pneumoniae* (81/220, 37%). CRPs were most frequently identified in urine, blood, and respiratory samples (Figure 1). Community-acquired infections were frequently diagnosed in patients infected with CRPs in our study (73% [161/220]), and most of the patients were admitted to the ICU (163/220, 74%). The in-hospital mortality rate was 28% (62/220) and 38% (62/163) in ICU admitted patients. PCR was carried out in 105 CRP; KPC (69%, 73/105) and VIM (37%, 39/105) were the most frequently identified mechanisms. Of the *K. pneumoniae* isolates with PCR assessment, 94% (33/35) had KPC and 3% (1/35) had VIM. In contrast, in *P. aeruginosa* isolates with PCR assessment, 53% (29/54) had KPC and 59% (32/54) had VIM. Seven (13%) patients infected with *P. aeruginosa* had both KPC and VIM genes identified.

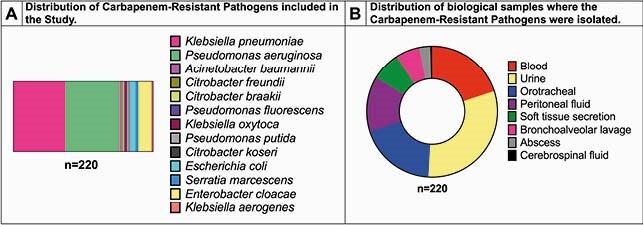

**Conclusion:**

The most frequently identified carbapenem-resistant pathogens in these two Colombian reference hospitals were *P. aeruginosa and K. pneumoniae*, with high mortality rates. KPC was the most commonly identified mechanism of carbapenem resistance in our cohort.

**Disclosures:**

**All Authors**: No reported disclosures

